# Promising peak flow diary compliance with an electronic peak flow meter and linked smartphone app

**DOI:** 10.1038/s41533-020-0178-y

**Published:** 2020-05-08

**Authors:** Thomas Antalffy, Anna De Simoni, Chris J. Griffiths

**Affiliations:** 1 Smart Respiratory Products Ltd, London, UK; 2 Asthma UK Centre for Applied Research, Institute of Population Health Sciences, Barts and The London School of Medicine and Dentistry, Queen Mary University of London, London, UK

**Keywords:** Diseases, Asthma

## Abstract

Peak expiratory flow (PEF) monitoring is recommended in the management of asthma. However, compliance is poor, and this is often attributed to the burden of measurement and recording. The Smart Peak Flow (SPF) device and app allow self-measuring and self-monitoring of PEF. Compliance with self-monitoring was promising in 399 UK users, calling for research to confirm these results and explore its potential as an intervention to improve self-monitoring.

## Introduction

The peak expiratory flow (PEF) is an objective measure of lung function that has been widely used in the diagnosis and monitoring of asthma^1^. Despite this, adherence levels to PEF monitoring have been shown to be as low as 9%^2^ and PEF “diary fabrication” as high as 60%^3^.

Patient education, regular reviews and feedback to patients^4^ improve adherence to PEF monitoring; however, the utility of PEF in clinical practice is hampered by a reliance on patients to manually document their PEF, leading to unreliable data recording and poor patient engagement. In view of this, the use of peak flow in clinical practice is not widespread. We hypothesized that an electronic PEF meter that automatically saves data onto a paired app will lead to improved adherence to PEF readings.

The Smart Peak Flow (SPF) device and app^5^ allow self-measuring and self-monitoring of PEF. To maximize adherence to PEF monitoring, additional app functionalities that users can select include: (1) PEF reminders, (2) immediate charting, (3) motivating messages, (4) challenges and rewards and (5) sharing of measurement results and charts with family members or health-care professionals via easily generated PDF of the measurements charts. The SPF can be purchased online by members of the public; the cost of the device is comparable with traditional PEF meters^6^.

A total of 750 free devices were supplied by the SPF UK distributor to 35 clinics directly or through clinicians attending UK annual respiratory conferences. In all, 250 SPF devices were kept by nurses, technicians and doctors to test, while 500 were offered to patients at clinicians’ discretion during outpatient appointments. One hundred and thirty-three of these patients subsequently downloaded the SPF app and measured at least one PEF.

SPF devices were distributed online through Kickstarter^7^ (a global crowdfunding platform) since 2017, while online sales started in February 2019, resulting in 266 UK individuals who purchased the device measured at least one PEF.

Of the 399 UK users who either bought or were supplied a free device, 64% were female, with an overall mean age across genders of 36.7 years (SD 17.3).

Between June 2018 and September 2019, users generated a total of 11,632 peak flow measurements.

**Fig. 1:**
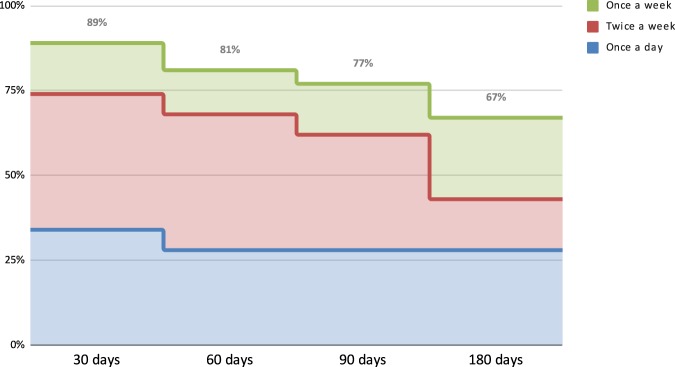
Smart Peak Flow compliance over time. After 6 months, 28% of users took at least a peak flow measure a day.

After 3 months, 32% of users took at least one peak flow measure a day, with 63% measuring their peak flow at least twice a week. After 6 months, 28% of users took at least a peak flow measure a day, with 67% measuring their peak flow at least once a week (Fig. 1).

Although no research evidence is available as yet, we speculate that these results could be partly explained by the higher motivation to self-monitor peak flow in users who self-funded the device or received support/encouragement to PEF monitoring by health-care professionals. Nonetheless, among participants who downloaded the SPF app, adherence is higher than reported rates to manual PEF meters^1,2^. Research is needed to confirm PEF adherence results, compare adherence before and after the introduction of the SPF device, the usability and acceptability of the SPF system for both patients with asthma and health-care professionals and its potential as an intervention to improve self-monitoring.

## Methods

Peak flow measurements are recorded using a turbine transduction system^8^ and backed up anonymously in secure cloud storage. In order to use the SPF system, users download the SPF app on their mobile phones and agree to the terms and conditions stated in the Privacy Policy. The Policy includes a statement informing participants about anonymized data collection of their app use, with the purpose of understanding the SPF impact on health through clinical research.

### Ethics declarations

The manuscript was examined by the Institutional Research Ethics Board and Caldicott Guardian at Queen Mary University of London and was exempt from full review.

### Reporting summary

Further information on research design is available in the Nature Research Reporting Summary linked to this article.

## Supplementary information

Reporting Summary

## Data Availability

The datasets generated during and/or analyzed during the current study are not publicly available due to patient consent restrictions, but are available from the corresponding author on reasonable request.
